# Early discharge (within 24–72 h) in low-risk AMI patients treated with PCI: feasibility and safety—Hajj study

**DOI:** 10.1186/s43044-020-00095-9

**Published:** 2020-09-07

**Authors:** Sheeren Khaled, Najeeb Jaha, Ghada Shalaby, Azmat Khadija Niazi, Faisal Alhazmi, Hadeel Alqasimi, Rahaf Abu Ruzaizah, Mryam Haddad, Mroj Alsabri, Heba Kufiah

**Affiliations:** 1 Benha University, Benha, Egypt; 2 King Abdullah Medical City, Muzdallfa Road, Makkah, Saudi Arabia; 3 Zagazig University, Zagazig, Egypt; 4 King Faisal Specialist hospital and research center, King Abdullah Medical City, Muzdallfa Road, Makkah, Saudi Arabia; 5 College of Medicine, Umm Al Qura University, Makkah City, Makkah 24353 Saudi Arabia

## Abstract

**Background:**

Shortening of the hospital stay in patients admitted with the diagnosis of acute myocardial infarction (AMI) has been observed within the last decades. Our center is the only cardiac center in the region providing tertiary care facility and hence receives all AMI patients deemed suitable for invasive assessment and management and this leads to huge required demand. Our aim is to assess feasibility and safety of the early discharge of selected proportion of AMI patients.

**Result:**

Out of 557 of patients presented with AMI and treated with percutaneous coronary intervention (PCI), 310 (56%) were discharged early. Men patients and pilgrims were more prevalent among the early discharge group. Early discharged patients had significantly less comorbidities compared to the other group of patients. Moreover, they presented mainly with ST-elevation myocardial infarction (*P* = 0.04) and treated more with primary percutaneous coronary intervention (PPCI) (*P* = 0.04). They had favorable coronary anatomy (*P* = 0.01 and 0.02 for left main and multi-vessel coronary artery disease, respectively), better hospital course, and higher left ventricular ejection fraction compared to non-early discharged patients (*P* = 0.006 and < 0.001 for pulmonary edema and left ventricular ejection fraction post myocardial infarction). Follow-up of those early discharged patients were promising as majority of them were asymptomatic (95%) and did well post-discharge.

**Conclusion:**

Our study demonstrated data that support safety of early discharge in a carefully selected group of AMI patients. Early but safe discharge may have a huge impact on increasing bed availability, reducing hospital costs, and improving patient’s satisfaction.

## Background

Shortening of the hospital stay in patients admitted with the diagnosis of acute myocardial infarction (AMI) has been observed within the last decades [1–3].

The current Guidelines of European Society of Cardiology for the management of acute myocardial infarction with ST-segment elevation, released in 2018, state that the selected patients may be considered (class of recommendation IIb) for early discharge (after approximately 72 h), if adequate follow-up is arranged [4]. This is also mentioned by some other observational studies [5–9].

Hajj is a great event and it is one of the five Islamic pillars. Millions of pilgrims from different countries of the world come to the Kingdom of Saudi Arabia for performing hajj. The overcrowding, hot climate, and huge physical stress expose the pilgrims to many health hazards. Cardiovascular disease has recently emerged as the leading cause of death during hajj [10, 11]. This makes great overload and burden all over the hospitals in Makkah region.

Our tertiary center is the only cardiac center in the region providing tertiary care facility including percutaneous coronary intervention (PCI) and coronary artery bypass grafting (CABG) and hence receives all AMI patients deemed suitable for invasive assessment and management and this leads to huge demand on cardiac services over the few weeks of hajj time. Reduced hospital stay has been proved to reduce the hospital burden and costs [12–15].

The aim of our study is to assess feasibility and safety of the early discharge of selected proportion of AMI patients. This facilitates establishment and implementation of the successful and safe early discharge program to enhance bed’s efficiency, improve occupancy rate and serve more patients during hajj crowdedness.

## Methods

This is a prospective, single-center study reviewing the clinical details of all AMI referred to our center for early revascularization and discharged during the two hajj seasons 2018 and 2019. It is designed to be a part of the standards of patient’s care, to investigate and improve quality of AMI management and outcomes among a diverse population; general and cath consents were taken and has received approval of the ethics committee/institutional review board of our institution.

As mentioned earlier, our center is the only cardiac center in Makkah with cath lab facilities, which provides invasive management of AMI, and we envisage capturing almost all AMI patients suitable for invasive management. Every year, our center receives about 1000 AMI patients; a quarter of them were admitted during the hot season in Makkah (2–3 weeks of hajj). Our study population included residence and hajj patients (who came from different countries and states with variable languages and educational backgrounds for doing their hajj). They were referred either from other primary, secondary, and Al-Mashaer hospitals or presented directly through our emergency department, and underwent coronary angiogram and PCI with drug eluting stents.

### Inclusion criteria

All patients admitted with established diagnosis of AMI (identified based on symptoms, ECG findings, and elevated troponin levels) during the few weeks of hajj seasons 2018 and 2019.

### Exclusion criteria

Patients presenting with AMI where coronary angiography was not done because of preference of the patient or due to any other reason and AMI patients admitted outside hajj seasons were not included as well.

The following data for each patient were collected:

1. *Demographics*. Age, gender, and status (residence/hajj)
2. *Risk factors*. Diabetes (DM), hypertension (HTN), smoking, dyslipidemia, presence of chronic kidney disease, old cerebrovascular accidents (CVA), chronic obstructive lung disease (COPD), ischemic heart disease (IHD), and previous PCI/CABG
3. *Clinical data*. Type of AMI are as follows:
  - ST-elevation myocardial infarction (STEMI) treated either with PPCI or with non-PPCI
  - Non-ST-elevation myocardial infarction (NSTEMI) with high-risk features (those who had recurrent chest pain, dynamic ECG changes, heart failure symptoms, and/or arrhythmias), which necessitate early revasculrization
    I History of pulmonary edema
    II Left ventricular ejection fraction (LVEF) in echocardiography
4. *Coronary procedure description and angiography findings*. Access site (femoral/radial), presence of left main disease (LM), single vessel disease (SVD), two or three vessel coronary artery disease (CAD), infarct-related artery (IAR), thrombus aspiration, and use of tirofiban/Aggrastat

Early discharge (24–72 h) was planned for selected patients who were fulfilled those criteria:

- AMI treated with successful PCI (TIMI flow III in infarct-related artery)
- Age ≤ 75 years
- Hemodynamic and rhythmic stability
- Absence of comorbidities, which require continuation of hospitalization
- Absence of contraindication of dual anti-platelet treatment
- Supposed cooperation, compliance, and adherence to medical treatment particularly DAPT (dual anti-platelet therapy)

Follow-up of early discharged patients with telephone communications within 15 days after discharge to check the safety of this strategy with collection of the following data:

- Alive/death with date
- Recurrent symptoms of ischemia
- Any bleeding
- Recurrent hospital admission

### Statistical analysis

Statistical analysis was performed by the use of the SPSS software package (SPSS Inc.; Chicago, IL); version 21.0. Continuous data were expressed as mean ± SD and compared using the Student *t* test. Categorical data were given as a percentage and compared with a Chi-square test. A *P* value < 0.05 was considered statistically significant.

## Results

**Fig. 1:**
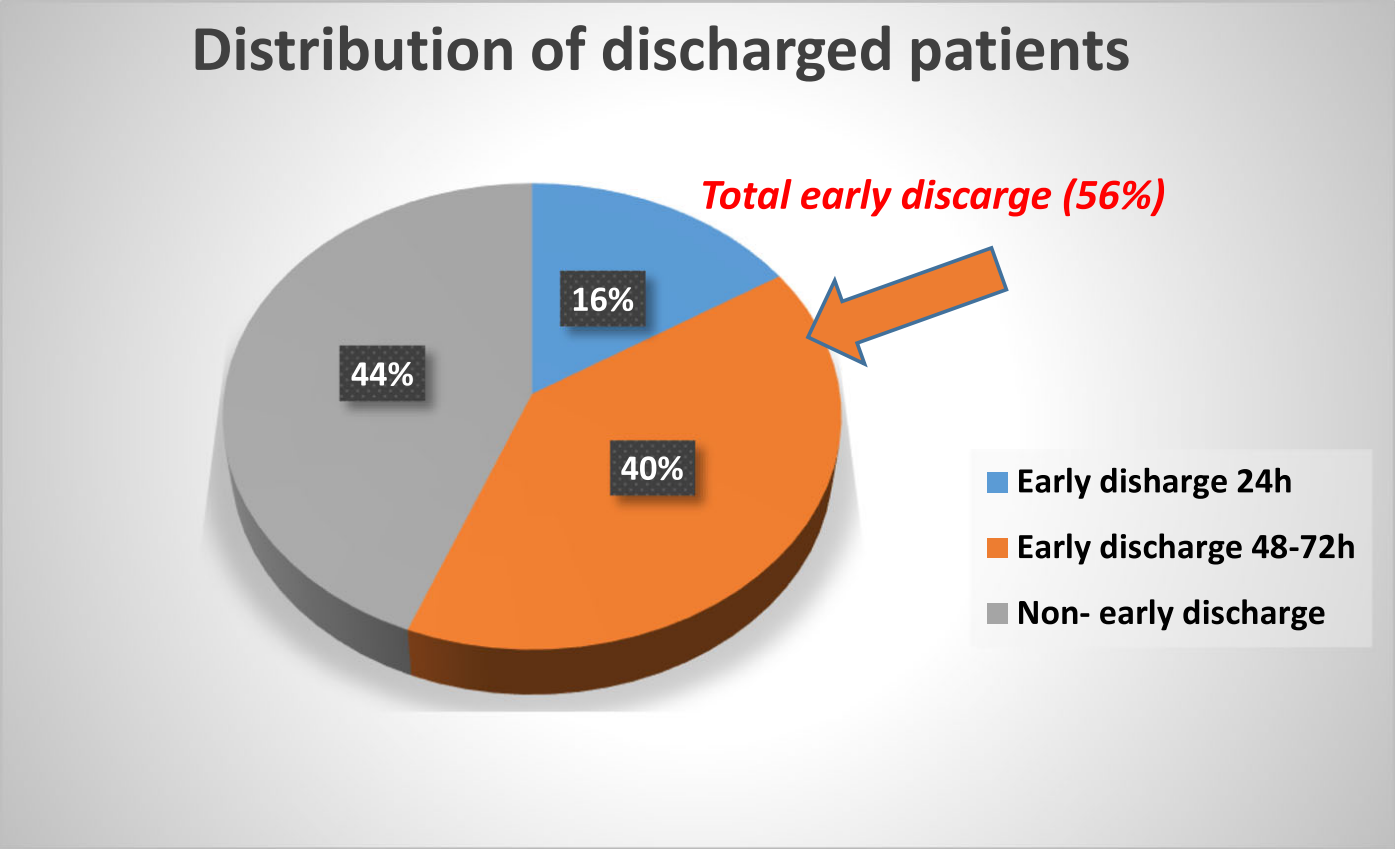
Distribution of the timing of discharge of the whole population

**Fig. 2:**
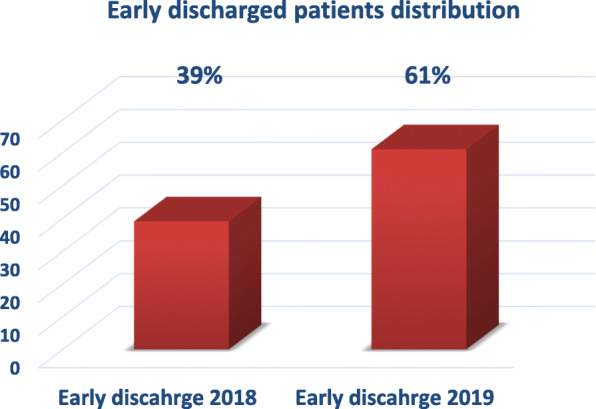
Early discharged patients distribution in hajj seasons 2018 and 2019

Out of 1972 AMI consecutive patients, five hundred fifty seven were treated with PCI during their admission in our center within the two hajj seasons (2–3 weeks of hajj) of the 2 consecutive years 2018 and 2019, respectively. Patients were distributed in to two groups: group I, 310 (56%) who were fulfilled the given early discharge criteria and discharged, and group II, 247 (44%) who were discharged late (after 72 h). From the whole discharged AMI patients in the study period, 89 (16%) were discharged within 24 h and 221 (40%) were discharged within 48–72 h from admission. One hundred twenty patients (39%) were early discharged in the hajj season 2018 while 190 (61%) were early discharged in the hajj season 2019 (Figs. 1 and 2).

### Demographics and baseline characteristics

Early discharge group of patients showed higher prevalence of both male gender and pilgrims (155 (50%) and 212 (68%) vs 87 (35%) and 146 (59%); *P* value < 0.001 and 0.02, respectively). They also showed less prevalence of DM (161 (52%) vs 148 (60%)), HTN (181 (58%) vs 158 (64%)), history of ischemic heart disease (55 (17%) vs 53 (21%)), and previous coronary revasculrization (27 (9%) vs 34 (14%)) compared to group II patients (Table 1).

**Table 1:** Baseline demographics and risk factors in the whole cohort and comparison of early discharge group versus non-early discharge group

Variables	Whole cohort, ***n*** = 557 (100%)	Early discharged patients (group I), ***n*** = 310 (56%)	Non-early discharged patients (group II), ***n*** = 247 (44%)	***P*** value
**Age, years**	56.75 ± 12.14	56.30 ± 12.17	57.31 ± 11.41	NS
**Male gender,** ***n*** **(%)**	242 (43)	155 (50)	87 (35)	< 0.001
**Pilgrims,** ***n*** **(%)**	358 (64)	212 (68)	146 (59)	0.02
**Hypertension,** ***n*** **(%)**	339 (61)	181 (58)	158 (64)	NS
**Diabetes,** ***n*** **(%)**	309 (56)	161 (52)	148 (60)	0.07
**Dyslipidemia,** ***n*** **(%)**	90 (16)	59 (19)	31 (13)	0.05
**Smoking,** ***n*** **(%)**	178 (32)	106 (35)	72 (29)	NS
**History of CAD,** ***n*** **(%)**	108 (19)	55 (17)	53 (21)	NS
**History of previous revascularization,** ***n*** **(%)**	61 (11)	27 (9)	34 (14)	0.03
**CKD,** ***n*** **(%)**	29 (5)	13 (4)	16 (6)	NS
**History of COPD,** ***n*** **(%)**	7 (1)	3 (1)	4 (2)	NS
**History of CVA,** ***n*** **(%)**	6 (1)	0	6 (2)	0.007

*CAD* coronary artery disease, *CKD* chronic kidney disease, *COPD* chronic obstructive pulmonary disease, *CVA* cerebro-vascular accident

### Clinical data

As shown in Table 2, STEMI patients treated with successful primary percutaneous coronary intervention (PPCI) were recorded higher among early discharge compared to non-early discharge group (251(81%) vs 176 (71%); *P* = 0.04). The early discharged patients had better hospital outcomes compared to the other group as they showed less prevalence of post myocardial infarction pulmonary edema (5 (2%) vs 15 (6%); *P* = 0.006) and higher LVEF (35.51 ± 17.89 vs 29.55 ± 19.84; *P* < 0.001). Otherwise, regarding the other treatment modalities, there were no detected significant differences between the two groups for the use of thrombolytic therapy and/or tirofiban post AMI treated with PCI.

**Table 2:** Clinical data in the whole cohort and comparison of early discharge group versus non-early discharge group

Variables	Whole cohort, ***n*** = 557 (100%)	Early discharged patients (group I), ***n*** = 310 (56%)	Non-early discharged patients (group II), ***n*** = 247 (44%)	***P*** value
**AMI presentation**				
**STEMI,** ***n*** **(%)**	451 (81)	267 (86)	184 (74)	0.04
**NSTEMI,** ***n*** **(%)**	106 (19)	43 (14)	63 (26)	
**PPCI,** ***n*** **(%)**	427 (77)	251 (81)	176 (71)	0.04
**History of thrombolytic therapy,** ***n*** **(%)**	24 (4)	16 (5)	8 (3)	NS
**Tirofiban use,** ***n*** **(%)**	104 (19)	57 (18%)	47 (19)	NS
**Pulmonary edema,** ***n*** **(%)**	20 (4)	5 (2)	15 (6)	0.006
**Post AMI-LVEF (%)**	32.85 ± 19	35.51 ± 17.89	29.55 ± 19.84	< 0.001

*AMI* acute myocardial infarction, *LVEF* left ventricular ejection fraction, *NSTEMI* non-ST-elevation myocardial infarction, *PPCI* primary percutaneous coronary intervention, *STEMI* ST-elevation myocardial infarction

### Coronary angiographic data

Radial axis was recorded as the higher selected approach among early discharged patients (222 (72%) vs 171 (69%); *P* = 0.003). Also, patients of group I with early discharge had favorable coronary artery disease anatomy as they showed less prevalence of both left main and three vessel disease in their coronary angiography compared to those of group II (7 (2%)and 55 (17%) vs 17 (7%) and 65 (26%); *P* = 0.01 and 0.02, respectively) (Table 3)

**Table 3:** Coronary angiography findings in the whole cohort and comparison of early discharge group versus non-early discharge group

Variables	Whole cohort, ***n*** = 557 (100%)	Early discharged patients (group I), ***n*** = 310 (56%)	Non-early discharged patients (group II), ***n*** = 247 (44%)	***P*** value
**Radial axis,** ***n*** **(%)**	393 (71)	222 (72)	171 (69)	0.003
**LM disease,** ***n*** **(%)**	24 (4)	7 (2)	17 (7)	0.01
**Three vessel disease,** ***n*** **(%)**	120 (22)	55 (17)	65 (26)	0.02
**LAD-IRA,** ***n*** **(%)**	310 (56)	178 (57)	132 (53)	NS
**Thrombus aspiration,** ***n*** **(%)**	43 (8%)	22 (7)	21 (9)	NS

*IRA* infarct-related artery, *LAD* left anterior descending artery, *LM* left main

### Follow-up data

**Fig. 3:**
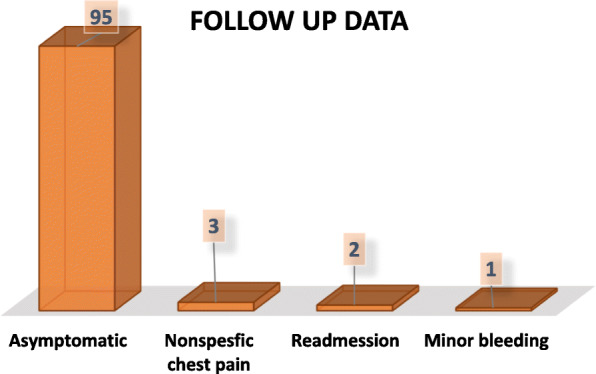
Follow-up data of early discharged patients

Among 310 early discharged patients, only 187 (60%) could be contacted by phone (with help of many translators of different languages) for follow-up within 15 days from the discharge date. They were questioned and found that 177 (95%) patients were totally asymptomatic, 6 (3%) had nonspecific chest pain which did not require any hospitalization, 2 (1%) had minor bleeding (epistaxis resolved spontaneously), and 2 (1%) re-admitted with heart failure symptoms. Those two patients re-admitted with heart failure had been found to have low ejection fractions with ischemic mild to moderate mitral regurge in their pre-discharge echocardiography and their follow-up echocardiography showed the same findings; however, they had mild chest infection, which predisposed to aggravation of heart failure (Fig. 3).

**Fig. 4:**
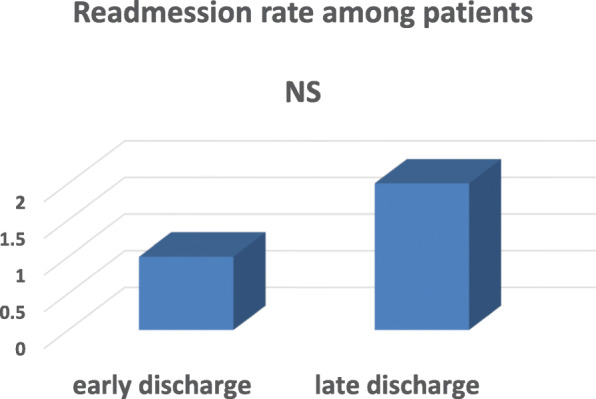
Comparison of readmission of early and late discharged patients

History of patient’s readmission was also used as indicator to assess safety of early discharge program implementation in our center and it was found to be non-significant between early and late discharge groups (2 (1%) vs 6 (2%), respectively) (Fig. 4). No recorded death among our early discharged patients.

## Discussion

The overview of most of literature investigating the issue of safety and feasibility of early discharge in AMI patients shows variable methodology and this explains that guidelines for early discharge are based just on limited data derived from randomized trials. The issue of early discharge was investigated in PRAGUE 5 study, which after pilot phase randomized 56 low-risk probands with STEMI, discharged even the next day after successful PCI. It was the first study in which mean length of hospital stay was shorter than 72 h [16]. Most of the studies concerned with early discharge developed practical score for risk stratification of their AMI patients to identify the patients with low risk of subsequent complications who do not require extensive in-hospital monitoring and observation [17–19].

It has been proven that substantial reduction in-hospital length of stay has been associated with reduction of in-hospital charge [12] with no increase in post-discharge mortality [1]. Some authors in previous studies concluded that patients with low risk of subsequent complications can be safely discharged within 2 days following primary PCI [5, 7–9], but it is important to focus on the fact that shorter hospital stay limits time for appropriate patient rehabilitation and education. Thus, the clinical follow-up post-early discharge should be carried out to assess safety.

Our center is the only cardiac center in the region providing tertiary care facilities and receives a huge number of AMI patients especially during the 2 to 3 weeks of hajj seasons, subsequently this puts a big burden for the required provided service and hospital costs. The primary aim of our study is to create and implement successful safe early discharge program to improve bed’s utilization efficiency and provide the best, safe, and maximum service for cardiac patients during over crowdedness of the hajj season. To the best of our knowledge, there are no similar studies conducted in our region concerned with this idea. We selected few weeks of each hajj season to conduct this study for many reasons: First, as previously mentioned, our cardiac center is the only center in the region of Makkah that has cath lab facilities and this required to provide tremendous tertiary care services to pilgrims and residents of Makkah during hajj season. Therefore, appropriate improvement of bed utilization is crucial with subsequent increase of hajj population in the successive years. Second, during this selected period, most of our AMI patients were pilgrims who had special ritual, religious emotions, and soul as most of them were doing their hajj for the first time having beliefs that longer hospital stay might reduce the opportunity to complete the hajj for which they were coming and persistently asking all the time for discharge. We believe that appropriate early post AMI discharge might grant their wishes provided it is totally safe. Third, during this short 2 weeks of hajj season additional two millions of population increase within Makkah city from pilgrimage and this requires a huge health service demand. Many facilities provided by the Ministry of Health and Hajj Committee during hajj seasons to provide a tremendous 24 h non-stop cardiac services included increasing manpower, raising number of working cath labs, improving working network, expanding all available services, and hence all that motivate the huge work and discharge of stable cases. We started implementation of early discharge program on hajj season 2018 and continued on 2019 to enlarge our sample size, which might help to gather more data and generalize our conclusion.

Our results concluded that those early discharged patients had higher percentage of pilgrims who were in high need for early discharge to compete their hajj pillars with their groups and return back to the their home countries safely. They also showed to have low-risk AMI features including less prevalence of cardiovascular risk factors and comorbidities, which all might predict safe early discharge post revascularization. Early revascularization with PPCI with appropriate target for DBT (door to balloon time) was recorded higher among those early discharged patients and this reflects higher quality of service provided by our center to AMI patients and could help in the process of early discharging them from the hospital. Clinical features and short-term outcome of AMI patients post event are crucial once decided the discharge process and these were followed carefully during our program implementation (early discharged patient had lower Killip class and higher post myocardial infarction LV ejection fractions). Reassuringly, most of our early discharged patients had favorable coronary artery disease anatomy (lower prevalence of both left main and multi-vessel disease) and this encouraged their early discharge process.

Follow-up data results were impressive as majority of our early discharged patients were totally asymptomatic (95%). Few percentages of those patients had non-serious symptoms and did not require any major intervention and this might reflect the early success of implementation of such program in our facility, which is considered the corner stone in the region.

Finally, our current experience reflects many advantages: great compensation of the huge required hospitalization burden; provision of the best, safe, and maximum service for cardiac patients during over crisis periods; lower hospital cost; and improvement of patient’s satisfaction.

### Limitation

Our study is limited to a single center and relatively small population number (short period selected only two hajj seasons) corresponds to the conclusions of mentioned studies. There are many factors explaining limitation of our follow-up data (as 40% of our early discharged patients lost their follow-up): language barrier, wrong written contact number in our records, non-attending calls, and higher percentage of those patients were pilgrims who returned back to their countries immediately after hajj without any follow-up here. We tried to support our results with some previous randomized studies with such recommended strategies and shorter length of hospital stay.

### Recommendation

We hope to reduce these limitations in future seasons by the following suggestions:

1. Suggested plan to establish proper educational program with help of the health promotion department supported with different language materials, which will be provided to those patients during their hospitalization regarding to their disease process, medication compliance and proper short-/long-term follow-up
2. Proper recording of correct contact numbers of the patients organized by our admission office and bed management departments
3. Motivation of the primary and secondary hospitals in the regions to conduct similar programs and possible organization of better follow-up protocols

## Conclusion

Our results provide preliminary data to support safety of early discharge in a carefully selected group of AMI patients while providing them dedicated telephone support and follow-up. Early but safe discharge may have huge impact on increasing patient’s satisfaction, bed availability (especially during hajj season) and reducing hospital costs. Furthermore, a larger multi-center study in the region has been encouraged to generalize our conclusion.

## Data Availability

The data that support the findings of this study are available on reasonable request from corresponding author but not publicly available due to privacy.

## Abbreviations

AMI: Acute myocardial infarction
ECG: Electrocardiogram
CABG: Coronary artery bypass grafting
CAD: Coronary artery disease
CKD: Chronic kidney disease
COPD: Chronic obstructive pulmonary disease
CVA: Cerebro-vascular accidents
DAPT: Dual anti-platelet therapy
DBT: Door to balloon time
DM: Diabetes mellitus
HTN: Hypertension
IHD: Ischemic heart disease
IRA: Infarct-related artery
LAD: Left anterior descending artery
LM: Left main
LV: Left ventricle
LVEF: Left ventricular ejection fraction
NSTEMI: Non-ST-elevation myocardial infarction
PCI: Percutaneous coronary intervention
PPCI: Primary percutaneous coronary intervention
STEMI: ST-elevation myocardial infarction
SVD: Single vessel disease
